# Visualizing the Effects of rTMS in a Patient Sample: Small N vs. Group Level Analysis

**DOI:** 10.1371/journal.pone.0015155

**Published:** 2010-12-08

**Authors:** Teresa Jacobson Kimberley, Richard P. Di Fabio

**Affiliations:** Department of Physical Medicine and Rehabilitation, University of Minnesota, Minneapolis, Minnesota, United States of America; Chiba University Center for Forensic Mental Health, Japan

## Abstract

The use of transcranial magnetic stimulation (TMS) to assess changes in cortical excitability is a tool used with increased prevalence in healthy and impaired populations. One factor of concern with this technique is how to achieve adequate statistical power given constraints of a small number of subjects and variability in responses. This paper compares a single pulse excitability measure using traditional group-level statistics vs single subject analyses in a patient population of subjects with focal hand dystonia, pre and post repetitive TMS (rTMS). Results show significant differences in cortical excitability for 4/5 subjects using a split middle line analysis on plots of individual subject data. Group level statistics (ANOVA), however, did not detect any significant findings. The consideration of single subject statistics for TMS excitability measures may assist researchers in describing the variably of rTMS outcome measures.

## Introduction

Transcranial magnetic stimulation (TMS) has become a widely used tool to assess cortical excitability in humans. A variety of TMS excitability measures exist, but universally, the amplitude of motor evoked potentials (MEPs) in muscles evoked by the TMS cortical stimulation is the outcome measure of interest that gives information about potential underlying neural mechanisms of changes that may occur [1]. When applied in a repetitive stream of pulses, repetitive TMS (rTMS) is used to induce inhibition or facilitation depending upon the frequency of the magnetic stimulation [2] and [3]. Other important factors are the duration and location of stimulation.

Recent work, however, has shown that response to rTMS can be variable between subjects. Ganitano and colleagues [4] report that a population analysis, in response to high and low frequency stimulation, followed the convention that high frequency rTMS increases and low frequency rTMS decreases cortical excitability. However, a post-hoc cluster analysis found two groups of subjects with opposite responses to the stimulation. Maeda and colleagues [5] report similar results, including an average frequency dependent increase in excitability, but found that each subject had a different modulatory effect on excitability given different rTMS stimulation frequencies. The reasons for these differences are not fully understood. It is hypothesized that intersubject anatomical factors related to sulci, gyri or interneuron orientation to the coil may be a critical factor [4]. Critical to elucidating these differences, however, is to apply statistical techniques that allow exploration of individual responses.

In addition to variability in response to an intervention, the variability of any outcome measure among groups of interest or between control and intervention phases within the same subjects is an important factor in brain research. High variability reduces reliability and is a barrier to findings of statistically significant differences. Variability of TMS excitability measures can be attributed to many factors including the intensity of stimulation [6], level of stimulation above or below motor threshold [7], and the failure to control for changes in the posttest motor threshold that occur with pretest rTMS exposure [3].

Attempts to minimize the variability of TMS outcome measures have been reported in the literature. Sommer and colleagues [8] reported the coefficients of variation of MEPs during single vs. paired pulse stimulation and concluded that single pulses yield more variability compared to paired pulses. Thus, suggesting that paired pulse outcome measures should be included in studies of excitability. Wassermann and colleagues [9] proposed the use of “recruitment curves” to control the variability of MEP amplitude assessment. These curves provide MEP output at a number of different, increasing, stimulation intensities and the slope of the resultant plot reportedly provides a “composite” and more reliable measure of cortical excitability. The authors suggest that evaluating differences in curve slope may be more sensitive than comparisons of MEP values at a given intensity. Recruitment curves however, still ignore individual responses and rely on averaged data from all subjects to demonstrate changes in cortical excitability. Any group-level analysis format has the potential to mask individual responders and confound the results by incorporating extreme amplitudes into the average amplitudes. This problem is magnified in studies with small N where small groups of people with rare neurological conditions are studied [e.g., focal hand dystonia (FHD) [10], [11]. Indeed it has been shown that subjects with FHD have higher variability in a TMS excitability measure than healthy subjects [12].Thus, the purpose of this paper is to present a method used in small N research called the *split-middle line*. This method is designed to evaluate the response of each subject and determine the statistical significance of change in MEP amplitudes pre and post rTMS. The outcome of this subject-by-subject analysis is compared to commonly used group-level statistics (repeated measures analysis of variance; RM ANOVA).

The demonstration sample studied here is a small group of people with FHD who received rTMS (specified in Methods). The development of FHD may be due to a lack of synaptic inhibition throughout the central nervous system [13]. People with FHD may suffer from hyper-excitability (decreased inhibition) of the corticospinal outputs to the affected hand [14]. Given the assumption of decreased inhibition in FHD, techniques that *facilitate* inhibition (low frequency rTMS) have a potential role in the treatment of the disorder [10].

## Methods

### Subjects

Five subjects with focal hand dystonia enrolled in this demonstration study (Table 1).

**Table 1:** Clinical and demographic subject information.

Case	Sex	Age	Handedness	Duration of symptoms	Diagnosis	Clinical Pattern
1	F	64	R	21 yr	WC R	R 3rd digit flexion during writing and typing
5	M	46	R	6 yr	WC R	R grip, wrist extensor spasms during writing/typing
9	M	42	R	14 yr	MD R	2nd/3rd digit spasm during typing and classical guitar playing
10	M	55	L	9 yr	MD L	L 2nd digit flexion playing piano
13	F	55	R	12 yr	WC R	R hand abnormal flexion/tremor during writing/mousing/pinching

R = right, L = left, WC = writer's cramp, MD = musician's dystonia, M = male, F = female.

### Ethics Statement

All subjects provided written informed consent prior to enrolling in the study according to the Declaration of Helsinki. The study was approved by the University of Minnesota General Clinical Research Center and Institutional Review Board. The funders had no role in study design, data collection and analysis, decision to publish, or preparation of the manuscript.

### rTMS

In this experiment, subjects were told that two levels of stimulation were being evaluated, a low level (sham) and high level (active). Each treatment protocol consisted of five consecutive days of rTMS application to the premotor cortex. A washout period of 9 days occurred between each sham and treatment phases. The phases were identical, except in the sham intervention, the figure-eight coil was positioned at 90 degrees from the cortex, thereby preventing the magnetic field from entering the head [15]. The subjects were unaware of the difference in coil orientation and still experienced the auditory inputs associated with the operation of the machine and felt the pressure of the coil against their head.

Subjects were seated comfortably semi-reclining in a chair and surface electrodes were affixed to the skin overlying the first dorsal interosseus muscle (FDI) of the involved hand in a belly/tendon montage. Electromyographic (EMG) signals were acquired at a sampling rate of 2560 kHz using a Cadwell Sierra EMG amplifier (Cadwell Laboratory, Washington) (sensitivity: 100* µ*v/div, filter: 20–2000 Hz). To find the optimal position for activating the FDI muscle, a 70-mm figure-of-eight TMS coil connected to a Magstim 200 Rapid magnetic stimulator (Magstim Co., Whitland, Dyfed, UK) was used. The coil was positioned with the handle directed posterolaterally 45° to the mid-sagittal line of the head over the approximate location of maximal sensitivity for FDI muscle activation (hotspot). Single-pulse magnetic stimuli were delivered manually until an MEP was elicited. This location was used to determine the resting motor threshold (rMT), defined as the minimum intensity required to elicit MEP amplitude *>*50* µ*Vpeak-to-peak in at least 3 of 5 trials in the resting target muscle [16]. The rMT was then used to determine stimulus intensity for single pulse assessment which was set to 130% of rMT. At rest, MEP data was collected for 5 single pulse stimulations applied at 0.1Hz to predetermined hotspot (gain: 200–500* µ*v/div filter: 20–2000 Hz). The outcome measure for cortical excitability therefore was the MEP of the FDI expressed in microvolts.

The rTMS intervention was provided with a Magstim Rapid2 magnetic stimulator (Magstim Co. LTD, Whitland, UK) connected to a 70-mm air-cooled coil (Numatic Int. LTD, Chard, UK) applied to the premotor cortex which has previously been defined as 1 cm medial and 2 cm rostral to the hotspot [17], [18], [19]. The active intervention consisted of 1 Hz rTMS at 90% of the subject's rMT, applied for 30 minutes (1800 pulses) to the premotor cortex contralateral to the tested finger.

### Experimental Design and Statistical Analyses

A single group cross over design was used to evaluate the effects of rTMS. All subjects received 5 single pulse measurements pre and 5 post application of sham or real rTMS. Assessments for cortical excitability were taken on day 1, 3, 5 of each phase (sham and intervention). This resulted in a total of 10 measurements at each day, or 30 in total.

Since the purpose of this paper was to compare and contrast “group level” statistics with “small N” individual statistical procedures, the following protocols were implemented.

#### Individual Statistics

An “A-B” design was selected for this demonstration because it has features (sham phase vs treatment phase) that are directly comparable to group level statistics. The A-B design has 2 phases; “A” is the sham phase and “B” is the intervention phase. There are many approaches to evaluating outcomes using small N research methodology [20], [21]. All of these approaches require a separate graph of each patient's outcome over time. For each subject's data plot, a split-middle line was constructed in the sham phase and then extended into the intervention phase. The split-middle line divides the data in sham phase into 2 equal parts and then calculates the median value for each half within the control data. This gives two points to form a line, which when extended into the treatment phase, creates a binomial distribution to test the significance of the distribution of points during the intervention [22].

The null hypothesis is that the data point distribution during intervention phase will show equal proportions above and below the extended split-middle line. The alternative one-tailed hypothesis is that the distribution of scores will occur prominently below the split middle line (showing that the intervention resulted in a reduction of cortical excitability). The procedures for implementing a split-middle test of significance for each subject in small N research are specified in detail elsewhere [22].

#### Group Level Statistics

A totally within group single factor repeated measures ANOVA (sham phase vs treatment phase) was used to determine the effect of rTMS on cortical excitability. The assumption of circularity (variances of differences between any two measurements within a subject are constant) was tested using Mauchly's test [23]. Three D'Agostino normality tests (skewness, kurtosis, and omnibus) were used to determine the normality of outcome score distributions [23]. In the case of non-normal score distributions, variable transformations were attempted (log and square root) in an attempt to normalize the score distributions [22].

## Results

### Cortical Excitability

#### Individual Subject Data Plots

**Figure 1:**
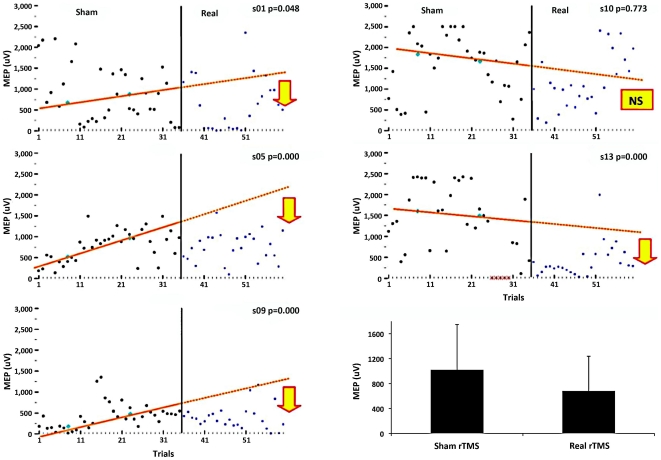
Motor evoked potentials of repeated single pulse stimulations at 130% resting motor threshold (repeated tests occurred at 0.1 Hz). Arrows indicate a significant decrease in cortical excitability during the intervention phase vs the sham phase for 4 subjects. Symbols on the x axis for s13 (жж) indicate missing data. Inset shows group level outcomes; means and standard deviations, N = 5.

Cortical excitability measured by MEP at 130% of rMT showed a statistically significant reduction in 4 out of 5 subjects during the intervention phase compared to the sham phase (**Fig. 1**). Visualization of these data on an individual level clearly demonstrates that the preponderance of the distribution of MEPs is below the extended split-middle line in subjects where cortical excitability was significantly reduced. The single subject with a non-significant reduction in MEP amplitude (s10) has a more diffuse and variable MEP output during the intervention phase (Fig. 1).

#### Group level Statistics

Results from the repeated measures ANOVA (RM ANOVA) for MEP data comparing sham vs intervention phases failed to produce a statistically significant main effect (F_1,4_ = 3.09, P = 0.154, ns). Mean MEP amplitudes are illustrated in **Figure 1** **-** **inset.** The assumption of circularity was verified. The data transformed by a square root were normally distributed for measures during the crossover phase but not the sham phase. The latter was resistant to transformation and neither transformation method created a normal distribution for the sham phase. Given that the RM ANOVA procedure is robust when the assumption of normality is partially violated [22] and considering that non-parametric methods do not address within subject variance, it was decided to proceed with the RM ANOVA as a relevant comparison of outcome against the single subject procedure. The RM ANOVA, however, did show a statistically significant interaction between session and subject (F_4, 280_ = 11.87, P<0.05). This means that the analysis identified some subjects who were “responders” vs “non-responders” by the *average performance* of each subject. These findings, however, were enhanced by applying SS procedures that allowed visualization of individual data points (not means) and illustrations of point-to-point variance.

## Discussion

In this report we have examined the difference between single subject analysis and group statistics in a single outcome measure in subjects with focal hand dystonia during sham and real rTMS intervention. We demonstrate that group level statistics failed to find a difference in cortical excitability across phases of the study. When evaluated with single-subject split middle test for significance, significance was found in 4/5 subjects. This type of analysis allows for a detailed analysis of subject-by-subject variation that is masked by group-level statistics.

The A-B design, used for this demonstration, is the weakest form of small N research because it is difficult to control for extraneous variables that might confound the outcome in the B phase (e.g., maturation-that is changes in subject behavior or response over time that is not related to the intervention)[20]. It was selected in this case, as it allowed direct comparison with a typical group design (sham and crossover). A stronger design would be to have a balanced, random distribution of A-B and B-A. In this case however, the risk to having the real intervention first was too great. A subject naïve to rTMS is less likely to identify the control rTMS as sham, and the duration of effect of the real intervention was unknown, thus there would have been a potential to have carryover into the sham phase if it was second. It is important to note that the results of single subject research cannot be applied to the population as a whole. It has been also been reported that the results of single subject research may be dependent upon the particular analysis format that is selected to judge the patient's response to the intervention [24]. The lack of consistency in outcome assessment using visual or statistical analyses applied to single subject graphed outcomes is a limitation of the single subject research approach to evaluating patient outcome over time.

The reason for lack of uniform change within this group is not the focus of the paper; however, a variety of factors may contribute to these findings. Individual subject characteristics may be a factor, including age, time of onset of symptoms, gender, or neuroanatomic variability. Issues of methods may also affect response. This could include the lack of neuronavigation to ensure repeatability of stimulation placement or the use of orthogonal stimulation instead of a sham coil in the sham phase.

Being mindful of the limitations, the consideration of single subject statistics for TMS excitability measures may assist researchers in describing the variably of rTMS outcome measures. Response to rTMS is known to be variable, with some subjects failing to demonstrate the expected excitability change following intervention [4], [5], [25] or in some populations the MEP may be difficult to achieve [26], [27], [28]. The source of individual variability in responsiveness is unknown, but has a significant impact on investigations. For example, in an investigation of a potential intervention for a patient population, if a subject does not respond to an intervention, one cannot determine if the lack of response was due to a general ‘rTMS unresponsiveness’ for that individual, or to a lack of efficacy of the intervention. In small n research, a few rTMS unresponsive subjects may cause a Type II error, of incorrectly accepting the null hypothesis. This could have major implications for “rTMS responsive” people who could potentially benefit from further investigation of the intervention. Some researchers attempt to control for this problem by including only known responsive subjects [29]. This is typically done by examining a ‘same day’ response to rTMS by determining an arbitrary amount in a given excitability measure that a given subject must change to be considered a “responder”. That subject is then included in the efficacy study if the change was adequate, but if the threshold of change in excitability is not met, they are excluded from participation. The limitation with any exclusion procedure is that the parameters of the “responsiveness test” are arbitrary and may not adequately characterize the subject's response to the intervention as a whole (e.g., lack of a response after one day may not mean that repeated interventions would fail to produce a response). It also fails to allow for a comprehensive evaluation of characteristics that may correlate with higher responsiveness and limits the ability to develop a predictive model.

Thus, it is proposed that small N statistics can be used as a means to accompany group-level analysis. This would serve to elucidate group responses as well as gain further understanding of why variable responses to an intervention occur within the sample.

## References

[1] Wassermann EM, Lisanby SH (2001). Therapeutic application of repetitive transcranial magnetic stimulation: a review.. Clinical Neurophysiology.

[2] Pascual-Leone A, Walsh V, Rothwell J (2000). Transcranial magnetic stimulation in cognitive neuroscience–virtual lesion, chronometry, and functional connectivity.. Curr Opin Neurobiol.

[3] Fitzgerald PB, Brown TL, Daskalakis J, Chen R, Kulkarni J (2002). Intensity-dependent effects of 1 Hz rTMS on human corticospinal excitability.. Clinical Neurophysiology.

[4] Gangitano M, Valero-Cabre A, Tormos JM, Mottaghy FM, Romero JR (2002). Modulation of input-output curves by low and high frequency repetitive transcranial magnetic stimulation of the motor cortex.. Clinical Neurophysiology.

[5] Maeda F, Gangitano M, Thall M, Pascual-Leone A, Maeda F (2002). Inter- and intra-individual variability of paired-pulse curves with transcranial magnetic stimulation (TMS).. Clinical Neurophysiology.

[6] van der Kamp W, Zwinderman AH, Ferrari MD, van Dijk JG (1996). Cortical excitability and response variability of transcranial magnetic stimulation.. J Clin Neurophysiol.

[7] Devanne H, Lavoie BA, Capaday C (1997). Input–output properties and gain changes in the human corticospinal pathway.. Exp Brain Res.

[8] Sommer M, Wu T, Tergau F, Paulus W (2002). Intra- and interindividual variability of motor responses to repetitive transcranial magnetic stimulation.. Clin Neurophysiol.

[9] Wassermann EM, Wedegaertner FR, Ziemann U, George MS, Chen R (1998). Crossed reduction of human motor cortex excitability by 1-Hz transcranial magnetic stimulation.. Neurosci Lett.

[10] Borich M, Arora S, Kimberley TJ (2009). Lasting effects of repeated rTMS application in focal hand dystonia.. Restorative Neurology and Neuroscience.

[11] Stinear CM, Byblow WD (2004). Impaired modulation of corticospinal excitability following subthreshold rTMS in focal hand dystonia.. Hum Mov Sci.

[12] Kimberley TJ, Borich MR, Prochaska KD, Mundfrom SL, Perkins AE (2009). Establishing the definition and inter-rater reliability of cortical silent period calculation in subjects with focal hand dystonia and healthy controls.. Neuroscience Letters.

[13] Berardelli A, Inghilleri M, Rothwell JC, Romeo S, Currà A (1998). Facilitation of muscle evoked responses after repetitive cortical stimulation in man.. Exp Brain Res.

[14] Quartarone A, Siebner HR, Rothwell J C (2006). Taskspecific hand dystonia: can too much plasticity be bad for you?. Trends in Neurosciences.

[15] Muellbacher W, Ziemann U, Boroojerdi B, Hallett M (2000). Effects of low-frequency transcranial magnetic stimulation on motor excitability and basic motor behavior.. Clin Neurophys.

[16] Rossini PM, Berardelli A, Deuschl G, Hallett M, Maertens de Noordhout AM (1999). Applications of magnetic cortical stimulation. The International Federation of Clinical Neurophysiology.. Electroencephalography & Clinical Neurophysiology - Supplement.

[17] Murase N, Rothwell JC, Kaji R, Urushihara R, Nakamura K (2005). Subthreshold low-frequency repetitive transcranial magnetic stimulation over the premotor cortex modulates writer's cramp.. Brain.

[18] Schluter ND, Rushworth MF, Passingham RE, Mills KR (1998). Temporary interference in human lateral premotor cortex suggests dominance for the selection of movements. A study using transcranial magnetic stimulation.. Brain.

[19] Fink GR, Frackowiak RS, Pietrzyk U, Passingham RE (1997). Multiple nonprimary motor areas in the human cortex.. Journal of Neurophysiology.

[20] Backman CL, Harris SR, Chisholm JM, Monette AD (1997). Single-subject research in rehabilitation: a review of studies using AB, withdrawal, multiple baseline, and alternating treatments designs.. Arch Phys Med Rehabil.

[21] Ottenbacher KJ (1986). Reliability and accuracy of visually analyzing graphed data from single-subject designs.. Am J Occup Ther.

[22] Portney L, Watkins M (2009). Foundations of Clinical Research: Applications to Practice..

[23] Hinze J (2007). NCSS Help System..

[24] Nourbakhsh MR, Ottenbacher KJ (1994). The statistical analysis of single-subject data: a comparative examination.. Phys Ther.

[25] Maeda F, Keenan JP, Tormos JM, Topka H, Pascual-Leone A (2000). Interindividual variability of the modulatory effects of repetitive transcranial magnetic stimulation on cortical excitability.. Experimental Brain Research.

[26] Moll GH, Heinrich H, Wischer S, Tergau F, Paulus W (1999). Motor system excitability in healthy children: developmental aspects from transcranial magnetic stimulation.. Electroencephalography & Clinical Neurophysiology - Supplement.

[27] Carey JR, Anderson DC, Gillick BT, Whitford M, Pascual-Leone A (2010). 6-Hz primed low-frequency rTMS to contralesional M1 in two cases with middle cerebral artery stroke.. Neuroscience Letters.

[28] Koh TH, Eyre JA (1988). Maturation of corticospinal tracts assessed by electromagnetic stimulation of the motor cortex.. Archives of Disease in Childhood.

[29] Duque J, Mazzocchio R, Stefan K, Hummel F, Olivier E (2008). Memory formation in the motor cortex ipsilateral to a training hand.. Cerebral Cortex.

